# Manganese Ferrite Nanoparticles Enhance the Sensitivity of Hepa1-6 Hepatocellular Carcinoma to Radiation by Remodeling Tumor Microenvironments

**DOI:** 10.3390/ijms22052637

**Published:** 2021-03-05

**Authors:** Sung-Won Shin, Kyungmi Yang, Miso Lee, Jiyoung Moon, Arang Son, Yeeun Kim, Suha Choi, Do-hyung Kim, Changhoon Choi, Nohyun Lee, Hee Chul Park

**Affiliations:** 1 Department of Radiation Oncology, Samsung Medical Center, Seoul 06351, Korea; camuserik@gmail.com (S.-W.S.); kyungmi.yang@samsung.com (K.Y.); arang.son@sbri.co.kr (A.S.); yeeun17.kim@sbri.co.kr (Y.K.); suha.choi@sbri.co.kr (S.C.); dohyung0.kim@sbri.co.kr (D.-h.K.); changhoon1.choi@samsung.com (C.C.); 2 Department of Medicine, Samsung Medical Center, Sungkyunwan University School of Medicine, Seoul 06351, Korea; 3 School of Advanced Materials Engineering, Kookmin University, Seoul 02707, Korea; dlalth0526@gmail.com (M.L.); wldud0869@kookmin.ac.kr (J.M.)

**Keywords:** radiotherapy, radiosensitization, nanoparticles, tumor hypoxia, tumor microenvironment

## Abstract

We evaluated the effect of manganese ferrite nanoparticles (MFN) on radiosensitization and immunologic responses using the murine hepatoma cell line Hepa1-6 and the syngeneic mouse model. The clonogenic survival of Hepa1-6 cells was increased by hypoxia, while being restricted by ionizing radiation (IR) and/or MFN. Although MFN suppressed HIF-1α under hypoxia, the combination of IR and MFN enhanced apoptosis and DNA damage in Hepa1-6 cells. In the Hepa1-6 syngeneic mouse model, the combination of IR and MFN notably limited the tumor growth compared to the single treatment with IR or MFN, and also triggered more frequent apoptosis in tumor tissues than that observed under other conditions. Increased expression of PD-L1 after IR was not observed with MFN alone or the combination of IR and MFN in vitro and in vivo, and the percentage of tumor-infiltrating T cells and cytotoxic T cells increased with MFN, regardless of IR, in the Hepa1-6 syngeneic mouse model, while IR alone led to T cell depletion. MFN might have the potential to overcome radioresistance by alleviating hypoxia and strengthening antitumor immunity in the tumor microenvironment.

## 1. Introduction

Radiation therapy (RT), along with surgery and systemic therapies, is an essential treatment modality for cancers [1,2]. In fact, advances in diagnostic imaging and RT techniques have led to improvements in clinical outcomes [3]. However, it is still possible to experience therapeutic failure, such as recurrence or metastasis, following RT.

The mechanisms underpinning tumor radioresistance are not yet fully understood. Some hypotheses to explain the emergence of radioresistance have been proposed, based on the characteristics of the tumor and its interaction with the tumor microenvironment (TME) [4]. Traditionally, researchers have focused on possible modalities to overcome the lack of therapeutic response, or on resistance related to genetic alterations in tumor cells, such as DNA mutations and aberrant RNA expression [5]. In contrast, the interactions between the TME and the immune system have garnered interest as novel, paradigmatic factors of tumor resistance [6,7]. The TME encompasses all the components surrounding the tumor, including immune cells, fibroblasts, and blood vessels, as well as signaling molecules and the extracellular matrix [8]. This environment and its changes are known to affect the therapeutic response and prognosis of tumors [9]. Interestingly, tumor hypoxia, which is one of the main factors influencing the response to RT, has been suggested to lead to an unfavorable immune microenvironment. Furthermore, RT might alter the interactions between tumor cells and their immune microenvironment [8]. Therefore, it is likely that promoting radiosensitization by targeting the hypoxic response of tumors would not only increase cell death during RT but also modulate the tumor microenvironment.

Nanotechnologies targeting various mechanisms have been applied to cancer treatment, both clinically and experimentally. For example, metal oxide nanoparticles (NPs), capable of generating oxygen in situ, have been developed and might be utilized as radiosensitizing agents [10,11]. Continuous oxygen generation by ferrite-based NPs has also been reported [12]. Despite the potential of NPs, only a few studies exploring their association with RT or tumor hypoxia have been conducted.

In the present study, we aimed to analyze the synergistic antitumor effect of a combined treatment with manganese ferrite NPs (MnFe_2_O_4_-NPs, MFN) and RT and demonstrate the high catalytic ability of MFN to trigger radiosensitization and immune-related TME changes in vitro and in vivo.

## 2. Results

### 2.1. Synthesis and Characterization of MFN

**Figure 1:**
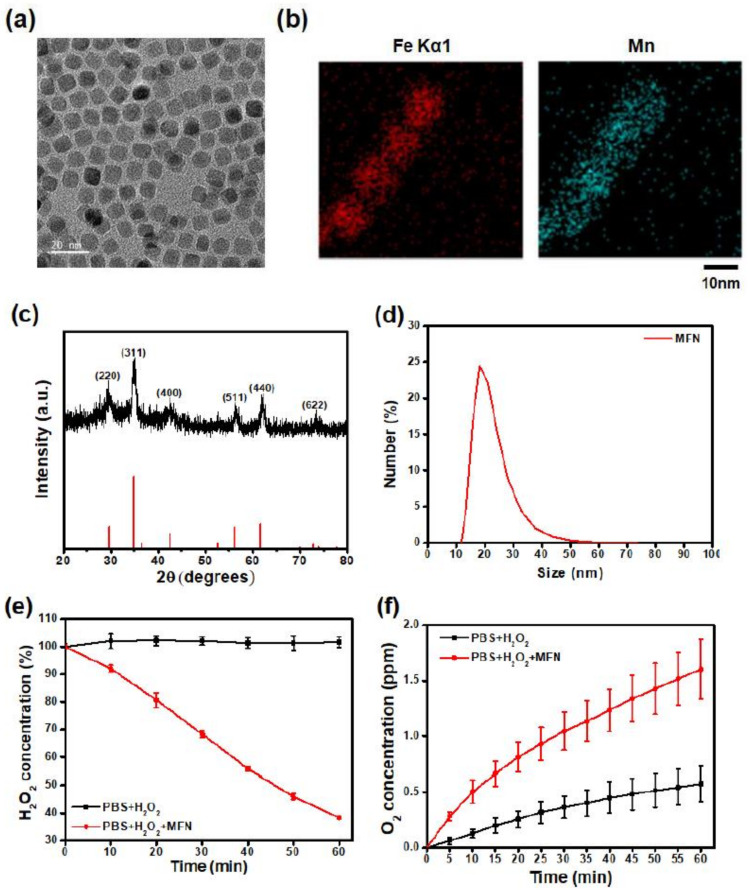
Synthesis of MnFe_2_O_4_ nanoparticles (MFN) and evaluation of their ability to degrade H_2_O_2_ and generate oxygen. (**a**) Transmission electron microscopy image of MFN. Scale bar: 20 nm. (**b**) Energy dispersive spectroscopic analysis of MFN. (**c**) X-ray diffraction pattern of MFN. (**d**) Hydrodynamic size distribution of MFN after PEGylation. (**e**) Decomposition of H_2_O_2_ by MFN via Fenton reaction. (**f**) Oxygen generation after treatment with MFN.

MFN were synthesized via thermal decomposition of iron acetylacetonate and manganese acetylacetonate in the presence of oleic acid. The transmission electron microscopy (TEM) image of MFN demonstrated a uniform size distribution (Figure 1a). The presence of iron and manganese ions was confirmed with energy-dispersive X-ray spectroscopy (EDS) (Figure 1b). The X-ray diffraction (XRD) pattern of MFN showed a spinel crystal structure of manganese ferrite (Figure 1c). The absence of impurity peaks indicated the absence of a secondary phase. Since the as-synthesized MFN were hydrophobic, oleic acid on the surface of the NPs was replaced with polyethylene glycol (PEG). After PEGylation, the MFN were colloidally stable in aqueous conditions. The hydrodynamic size measured with dynamic light scattering (DLS) was 21 nm (Figure 1d). Such an increase in size, compared to that observed in TEM images, can be attributed to the chain length of PEG and the hydrated layer around the NPs. The catalytic effect of MFN was evaluated by measuring the H_2_O_2_ concentration after the addition of MFN. Within 1 h, 62% of the H_2_O_2_ was degraded by MFN (Figure 1e). The oxygen concentration was measured to investigate the oxygen generation ability of MFN. Dissolved oxygen was removed by argon bubbling prior to MFN addition. A significant increase in oxygen concentration was detected along with the degradation of H_2_O_2_ by MFN (Figure 1f). Since MFN were not degraded during the reaction, the color of the solution did not change.

### 2.2. Effects of MFN on Cell Proliferation and Radiosensitization In Vitro

**Figure 2:**
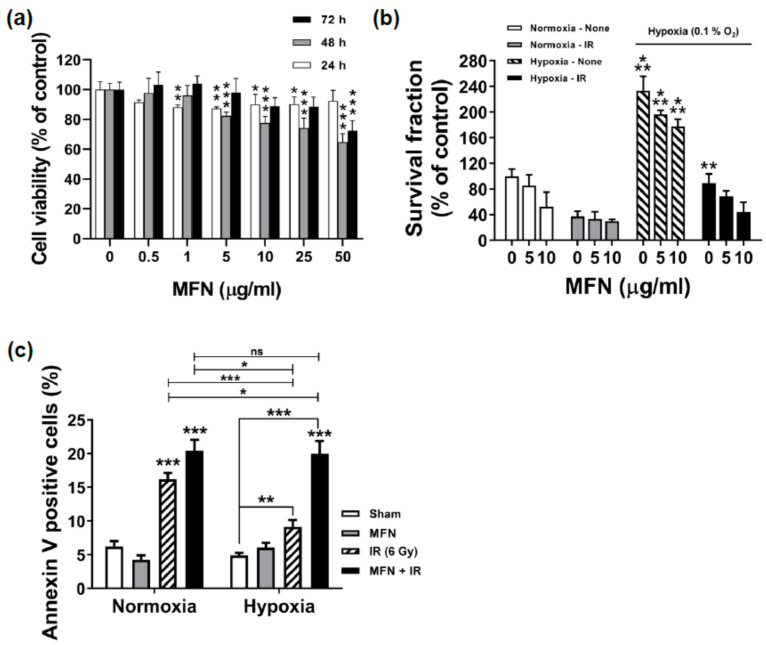
Effects of MnFe_2_O_4_ nanoparticles (MFN) on the viability and radiosensitization of murine hepatoma Hepa1-6 cells. (**a**) Concentration- and time-dependent cytotoxicity of MFN. Cell viability was determined using the CCK-8 assay. (**b**) Radiosensitizing effect of MFN under hypoxia. Clonogenic survival assays were performed as described in the Materials and Methods. Hepa1-6 cells were pre-incubated with 5 or 10 µg/mL of MFN for 3 h, and then subjected to a 6-Gy irradiation under normoxic or hypoxic conditions. (**c**) Promotion of apoptosis by MFN under hypoxia. Flow cytometry of Annexin V-stained cells was performed for the analysis of apoptosis. The values represent the mean ± standard deviation (SD); * *p* < 0.05; ** *p* < 0.01; *** *p* < 0.001; ns, not significant.

To evaluate the dose-dependent cellular toxicity of MFN, the Cell Counting Kit-8 (CCK-8) assay was performed (Figure 2a). A slight decrease in cell viability was observed at 24 h; this stabilized at approximately 90% of that of the control cells in response to MFN concentrations ranging from 1 to 50 µg/mL. At 48 h, 5 µg/mL or higher concentrations of MFN triggered an additional decrease in cell viability in a dose-dependent manner; nevertheless, a cell viability of over 80% was sustained at concentrations up to 5 µg/mL. At 72 h, cell viabilities were similar or higher compared to those observed at the same concentrations of MFN at 24 or 48 h; this effect might have been caused by cell proliferation. Notably, for MFN concentrations up to 5 µg/mL, cell viability at 72 h was nearly 100% that of the control.

To confirm the radiosensitizing effect of MFN under hypoxia, clonogenic survival assays were performed (Figure 2b). A dose-dependent effect of MFN was observed under all conditions; in particular, the cells treated with 5 µg/mL of MFN demonstrated higher survival than those receiving 10 µg/mL of MFN. Survival fractions under hypoxia were typically higher than those under normoxia; however, the combination of ionizing radiation (IR) and MFN dramatically decreased clonogenic survival even under hypoxia. Based on the results of the cell viability and clonogenic survival assays, an MFN concentration of 5 µg/mL was selected for further steps. The effect of MFN on radiation-induced apoptosis was analyzed using flow cytometry with Annexin V staining (Figure 2c). Under hypoxia in the absence of MFN treatment, the rate of apoptosis was significantly lower after IR than that observed under normoxia (*p* < 0.001, Figure 2c). However, after treatment with IR and 5 µg/mL of MFN, the rate of apoptosis recovered to levels similar to those observed under normoxia.

**Figure 3:**
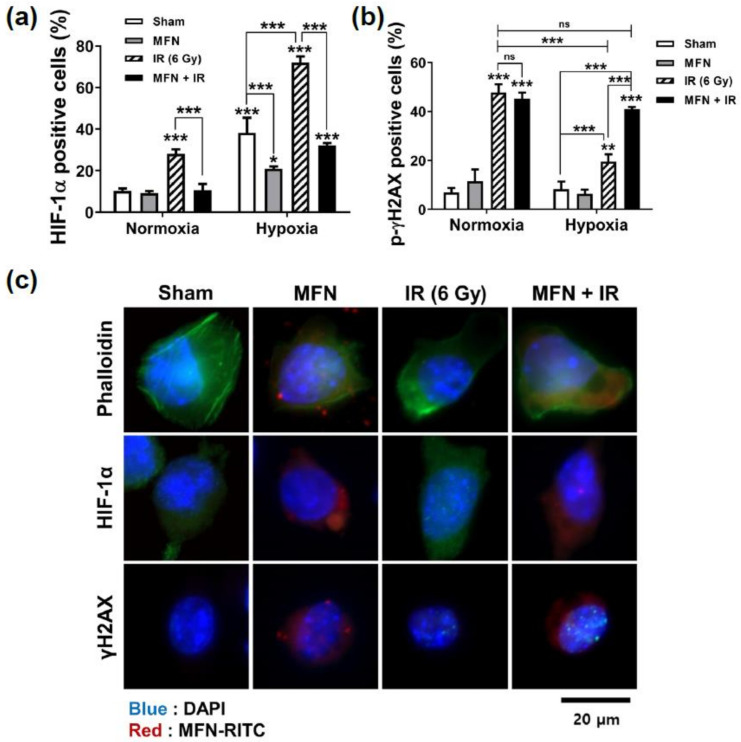
Effects of MnFe_2_O_4_ nanoparticles (MFN) on HIF-1α expression and DNA damage following ionizing radiation (IR). (**a**) Increased HIF-1α expression under hypoxia was suppressed by MFN treatment. Flow cytometry was performed as described in the Materials and Methods. (**b**) Decreased DNA damage under hypoxia was recovered by MFN treatment. (**c**) Representative immunofluorescent images of HIF-1α and γH2AX. Blue: DAPI; red: MFN; green: phalloidin, indicating HIF-1α and γH2AX. The values represent the mean ± SD; * *p* < 0.05; ** *p* < 0.01; *** *p* < 0.001; ns, not significant.

Next, the effect of MFN on radiation-induced DNA damage was determined with flow cytometry and immunofluorescence imaging (Figure 3). Flow cytometric analysis revealed that hypoxia inducible factor 1, alpha subunit (HIF-1α), a key marker of hypoxia, was highly expressed under hypoxic conditions, as expected; however, a significant difference in HIF-1α levels was observed upon treatment with MFN, regardless of IR administration (Figure 3a). Furthermore, under hypoxia, the expression of HIF-1α after IR without MFN treatment was higher than in any other condition. However, the expression of HIF-1α decreased significantly upon combined IR and MFN treatment. IR-induced DNA damage was assessed by counting the cells positive for gamma histone 2AX (γH2AX), a marker of DNA double-stranded breaks (Figure 3b). Hypoxia significantly decreased the γH2AX-positive cell population after IR compared to that observed under normoxia. However, the γH2AX levels were recovered to those observed under normoxic conditions upon combined treatment with MFN and IR. Immunofluorescence images of HIF-1α and γH2AX expression under hypoxic conditions (Figure 3c) showed that MFN relieved hypoxia and enhanced IR-induced DNA damage (Figure 3c), which was consistent with the flow cytometry data (Figure 3a,b). In particular, HIF-1α levels decreased in MFN-treated cells, while γH2AX nuclear foci increased in the cells co-treated with MFN and IR, compared to those in the cells treated with IR alone.

### 2.3. Effects of MFN on Tumor Radiosensitization In Vivo

**Figure 4:**
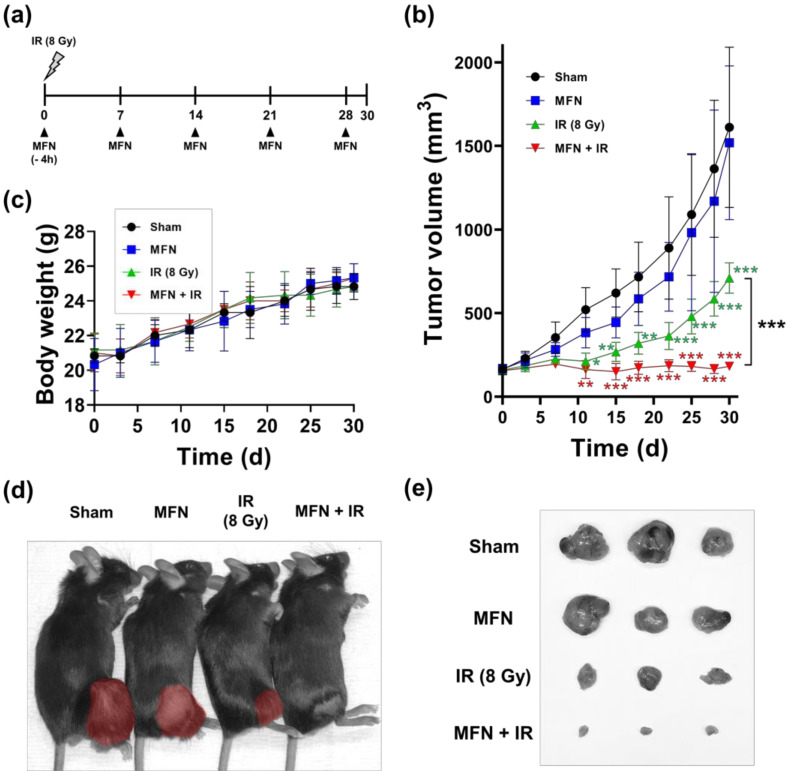
In vivo tumor radiosensitization by MnFe_2_O_4_ nanoparticles (MFN) in a Hepa1-6 syngeneic mouse model. (**a**) Scheme of the experimental procedures carried out using the mouse model. One fraction of the 8-Gy radiation dose was administered at the right hind leg where Hepa1-6 cells were implanted, and/or the MFN were repeatedly injected intratumorally. (**b**) Growth patterns of tumors in terms of tumor volume. (**c**) Changes in the body weight of mice during the experiment. (**d**) Macroscopic features of mice and implanted tumors (brown) at the end of the experiment. (**e**) Sizes of tumor tissues collected after euthanizing the mice on day 30. The values represent the mean ± SD; * *p* < 0.05; ** *p* < 0.01; *** *p* < 0.001; two-way ANOVA (*n* = 7).

The in vivo radiosensitizing effect of MFN was evaluated using a C57BL/6 mouse model implanted with Hepa1-6 cells (Figure 4a). When the tumors became palpable, the mice were randomized into four groups, namely, the sham control (treated with PBS), MFN (treated with 200 ng of MFN once a week), IR (irradiated with 8 Gy), and IR+MFN (treated with both MFN and IR). In a tumor growth assay, tumor volume in the IR group was significantly reduced compared to that in the control group (*p* < 0.001), and the combination of IR and MFN was more effective than IR alone (*p* < 0.05; Figure 4b). The mice demonstrated stable weight gains (Figure 4c) under all experimental conditions, even under IR and/or MFN treatment. Nevertheless, at the end of the experiment, sizes of the implanted tumors were remarkably different (Figure 4d,e). Tumors collected after IR treatment were smaller than those observed in the control or MFN groups, while those collected after co-treatment with IR and MFN were the smallest.

**Figure 5:**
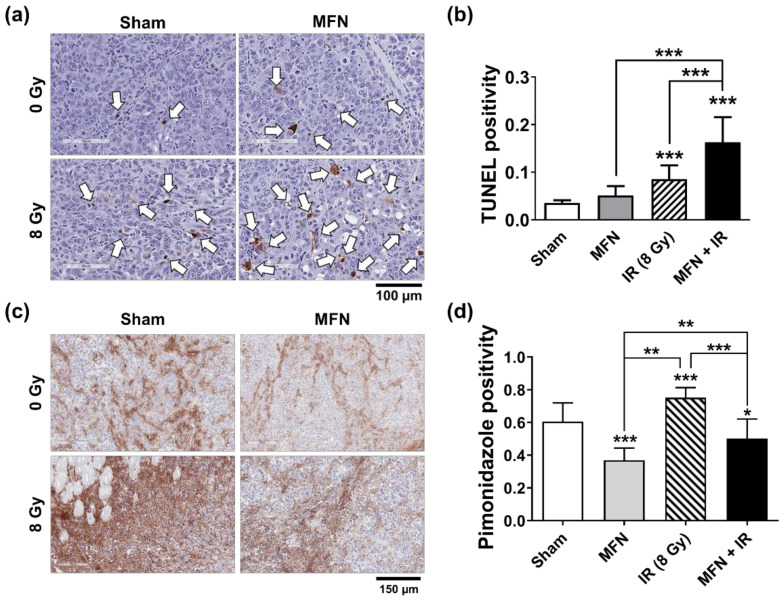
In vivo assessment of apoptosis and hypoxia status in Hepa1-6 tumor tissues following treatment with ionizing radiation (IR) and/or MnFe_2_O_4_ nanoparticles (MFN) in a mouse model. (**a**) Representative TUNEL staining images of the Hepa1-6 tumor tissues. Tumors were harvested on day 30. The white arrows indicate TUNEL-positive cells. (**b**) Quantitative comparison of TUNEL positivity among the experimental groups. The combination of IR and MFN significantly promoted apoptosis in tumor tissues. (**c**) Representative images of pimonidazole staining (brown color) showing an MFN-dependent decrease in hypoxia in Hepa1-6 tumor tissues. (**d**) Quantitative comparison of the pimonidazole levels among the experimental groups. The values represent the mean ± SD; * *p* < 0.05; ** *p* < 0.01; *** *p* < 0.001.

The rate of apoptosis in Hepa1-6 tumor tissues was measured using the terminal deoxynucleotidyl transferase dUTP nick end labeling (TUNEL) assay (Figure 5a). IR promoted apoptosis in tumor tissues, and the combination of IR and MFN triggered apoptosis rates twice as high as those observed without MFN treatment (Figure 5b). A hypoxia-specific reagent, pimonidazole, was used to assess hypoxia in the tumor tissues (Figure 5c). In the groups treated with MFN, pimonidazole levels were lower than those observed in the group treated with IR only (Figure 5d).

### 2.4. Effects of MFN on Radiation-Induced Immune Modulation

**Figure 6:**
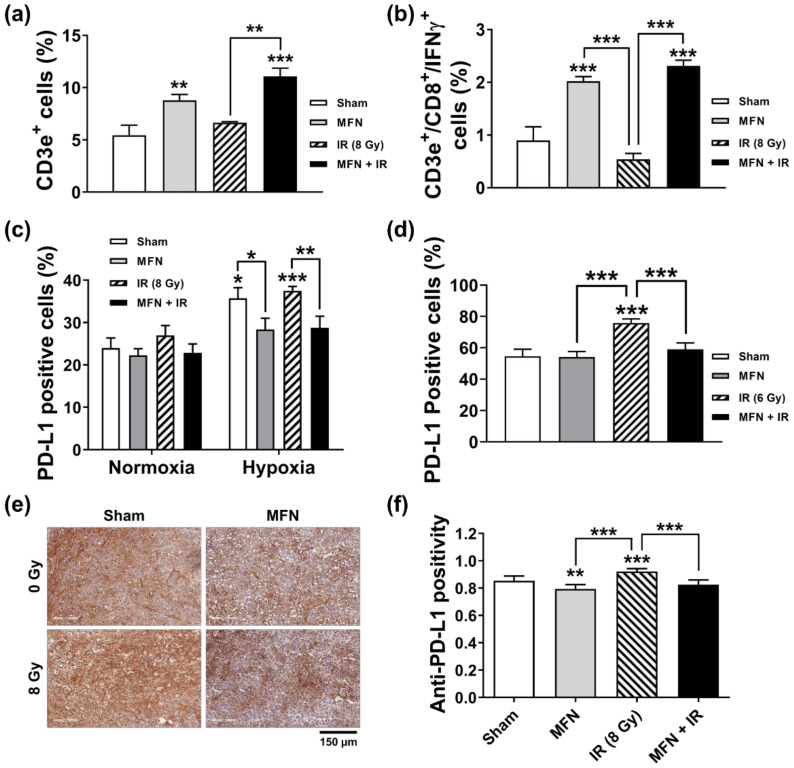
Modulation of T-cell infiltration and PD-L1 expression by MnFe_2_O_4_ nanoparticles (MFN). (**a**) MFN promoted the infiltration of CD3e+ T cells in tumor tissues harvested on day 30. (**b**) MFN increased the percentage of cytotoxic CD3e+/CD8+/IFNγ+ T cells following treatment with 8 Gy of ionizing radiation (IR) and intratumoral injection of MFN. (**c**) Flow cytometric analysis showed that MFN suppressed the hypoxia-induced PD-L1 expression in Hepa1-6 cells. Hepa1-6 cells were pre-incubated with 5 μg/mL of MFN under hypoxic or normoxic conditions, followed by 8 Gy of IR. (**d**) Flow cytometric analysis showed that MFN suppressed the radiation-induced PD-L1 expression in cells collected from tumor tissues. (**e**) Immunohistochemical analysis of PD-L1 expression in tumor tissues. (**f**) Comparison of PD-L1 positivity in the tumor tissues among the experimental groups. The values represent the mean ± SD; * *p* < 0.05; ** *p* < 0.01; *** *p* < 0.001.

Next, we tested the effect of combined treatment with IR and MFN on the tumor immune microenvironment. The proportion of tumor-infiltrating T lymphocytes and the expression of programmed cell death 1 ligand 1 (PD-L1) were analyzed using flow cytometry. Treatment with MFN, with or without IR, increased the percentage of total CD3e+ T cells infiltrating the tumor tissues, compared to the control (Figure 6a). Tumor-infiltrating T cells were statistically more abundant in the IR + MFN group than in the IR group (*p* < 0.01). In addition, more potent stimulation of cytotoxic CD3e+/CD8+/IFNγ+ T cells was observed after treatment with MFN, with or without IR administration (Figure 6b). In the Hepa1-6 cells cultured in vitro, PD-L1 expression was upregulated under hypoxia compared to normoxia, but such upregulation was suppressed by MFN, regardless of IR (Figure 6c). Flow cytometric analysis of cells from tumor tissues showed that the IR-stimulated PD-L1 expression was suppressed by MFN treatment (Figure 6d). Immunohistochemical (IHC) analysis of PD-L1 in the tumor tissues yielded results consistent with those of flow cytometry (Figure 6e,f).

## 3. Discussion

The immune system and TME have recently emerged as new targets for cancer treatment [13]. In particular, hypoxic conditions alter the composition of the TME, thereby affecting the immune response against cancer [14]. For example, HIF-1α expression influences the expression of molecules such as PD-L1, leading to the immune escape of tumor cells [15,16]. Hypoxia not only directly affects tumor-infiltrating immune cells but also indirectly alters the properties of endothelial cells through the recruitment of immunosuppressive cells, such as regulatory T cells and myeloid-derived T cells. Therefore, suppressing hypoxia is a promising way to improve the effectiveness of cancer treatment by remodeling the TME.

In radiation oncology, hypoxia is a traditional but still challenging subject. Reduced oxygen diffusion in the tumor is attributed to the long distance between cells and vessels and to the lack of mature vasculature due to abnormal rapid growth of the tumor [17,18]. Hence, DNA damage and cell death, typically observed after radiation treatment, occur less frequently under such conditions, resulting in a less marked therapeutic response. Many strategies for overcoming hypoxia have been examined so far. The following strategies demonstrated positive clinical outcomes: direct targeting of hypoxic cells, reducing oxygen dependence, enhancing oxygen delivery through the blood, or the use of oxygen analogs [17,19]. The evidence for the benefit of counteracting hypoxia has been evaluated to be relatively strong in some meta-analyses [20]. However, these strategies have been poorly applied in the clinic, and their effect on the TME has rarely been studied.

Radiation therapy itself might alter tumor immunity, thereby affecting the tumor response [8]. First, HIF-1α expression increases after RT. HIF-1α is related to angiogenesis, cell proliferation and survival, recurrence, and metastasis [21]. In addition, RT can deplete or exhaust the pool of infiltrated T cells, leading to an ineffective immune reaction [8]. Indeed, CD8+ T cells play important roles in the tumor and TME, and their tumor infiltration is considered a positive prognostic factor in cancer immunology [22]. However, RT can damage infiltrated T cells because lymphocytes are the most sensitive cells to RT. Furthermore, the expression of PD-L1 is increased in tumor cells after RT [23], and excess PD-L1 affects the number of T cells. Thus, co-administration of immune-checkpoint inhibitors, including anti-PD-1 or anti-PD-L1 antibodies, is considered effective for reinforcing anti-tumor immune responses during RT [24,25].

In this study, the application of MFN demonstrated the ability to overcome these immunogenic disadvantages arising from RT, and to remodel the TME favorably. We observed that mice treated with MFN only, IR only, and a combination of IR and MFN showed a progressive improvement in tumor response to RT. However, total and cytotoxic T-cell infiltration were enhanced by MFN treatment, regardless of IR. These results indicated that oxygen supply from MFN can alter the immune system in the TME, allowing more rapid recruitment of activated T cells even after RT. Since our experimental approach focused on the short-term tumor response to IR and MFN, it is difficult to explain or predict long-term effects of MFN supplementation. However, it is very likely that MFN could prevent tumor recurrence or metastasis after RT, as they suppressed HIF-1α expression and reversed immune suppression.

A variety of mechanisms have been reported to overcome the underlying mechanisms of hypoxia, and nanotechnology is an actively developing field with ongoing experimental research [8,26]. Metal-based NPs have been recognized as radiosensitizers, which help DNA damages by direct or indirect process [27]. High atomic number (Z) materials, such as gold, were expected to increase both the physical dose and chemical contribution by electron emission. As another mechanism for increasing O_2_ and reactive oxygen species, metal oxide NPs, including MnO_2_, a highly functional contrast agent [28], have been proven to work effectively against radioresistance, owing to their catalytic activity towards H_2_O_2_ [10,11]. Furthermore, some studies suggested that MnO_2_ NPs could affect the immune response and the abscopal effect of IR [29]. MFN are also known as strong catalysts for the Fenton reaction [30] and are expected to release a sustained supply of oxygen together with abundant active hydroxyl radicals generated from the catalytic transition of Fe^2+^ to Fe^3+^ and Mn^2+^ to Mn^3+^ during H_2_O_2_ scavenging [12]. Unlike MnO_2_, which is degraded after the catalytic reaction, the ions of MnFe_2_O_4_ can be recycled [31]. By exploiting this property, the present study showed the potential of MFN to overcome hypoxia and achieve a better response to RT, when combined with IR. Injected MFN can deliver oxygen directly to the center of large tumors independent from vessel transport. Interestingly, tumor cells are characterized by a significantly higher concentration of H_2_O_2_ than that of normal cells, and this is even higher in more aggressive tumors [32]. In fact, metabolically active cancer cells have an imbalanced redox status and accumulate H_2_O_2_ through the conversion of superoxide radicals by superoxide dismutase in the mitochondria [33]. Therefore, we propose that, by utilizing the high concentration of H_2_O_2_ present within tumors, MFN could alleviate hypoxia and overcome radioresistance in aggressive cancers. Although NPs represent a remarkable technology, there are limitations associated with their clinical use. In terms of RT, the first and only reported clinical trial using therapeutic NPs has been attempted in sarcoma, which is one of the tumors most resistant to therapy [34]. Here, the authors assumed that the high Z material used in the study (HfO_2_) would release a large number of electrons when hit by high-energy X-rays. The study showed a positive effect of HfO_2_ NPs in terms of survival after R0 resection (77% vs. 64%, *p* = 0.042) in a randomized phase II–III trial, without serious additional side effects [35]. HfO_2_ NPs were injected into the tumor directly; such a procedure is in theory clinically safe, as the NPs were eventually removed by surgery. Similarly, prior to clinical application, the biological stability and safety of nanomaterials should be confirmed. In this regard, there are few studies on MnO_2_ or MnFe_2_O_4_ NPs. Manganese ferrite is nearly non-toxic to cells; however, inorganic manganese materials carry a risk of triggering various types of toxicity [36,37]. In this study, rare complications were expected, as a small amount of MFN was injected directly into the tumors. Consistently, the growth of mice seemed normal. Despite the excellent therapeutic effect of MFN, preclinical studies examining their stability, for instance by dynamic imaging after injection, leakage to blood, or liver uptake, as well as clinical trials, to demonstrate their safety and therapeutic effectiveness, must be conducted. Based on these results, we expect that NPs with high catalytic functionality, such as MFN, may be used as radiosensitizing and immune-modulating agents for cancer treatment.

## 4. Materials and Methods

### 4.1. Synthesis and Characterization of MFN

MFN were synthesized via thermal decomposition of iron acetylacetonate and manganese acetylacetonate according to a previously reported method with minor modifications [38]. Iron acetylacetonate (1.5 mmol, 97%, Sigma-Aldrich, St. Louis, MO, USA) and manganese acetylacetonate (1 mmol, Sigma-Aldrich) were added to a mixture composed of oleic acid (10 mmol, 90%, Sigma-Aldrich) and benzyl ether (50 mL, 98%, Sigma-Aldrich) at room temperature. The resulting solution was heated to 60 °C and degassed for 1 h. Subsequently, the mixture was heated to 290 °C at a constant rate of 20 °C per minute and maintained at temperature for 30 min. After cooling the solution to room temperature, ethanol and methanol were added, and the solution was centrifuged at 5000 rpm for 15 min to precipitate the NPs. After several washing steps, the separated NPs were dispersed in chloroform. To exchange the ligand, 2-bromo-2-methylpropionic acid (BMPA, 1 g, Sigma-Aldrich) and citric acid (0.1 g, Sigma-Aldrich) were dissolved in a mixture of chloroform (12 mL, Samchun, Seoul, Korea) and N,N-dimethylformamide (DMF, 15 mL, Samchun). Subsequently, 60 mg of oleic acid-capped MFN was dispersed in this solution, which was then stirred overnight at room temperature. BMPA-capped MFN were retrieved after several steps of washing with ethanol and were re-dispersed in ethanol. For the PEGylation of MFN, 60 mg of methoxy polyethylene glycol amine (mPEG-AM, MW = 2000 Da, Sunbio Inc., Gunpo, Korea) was dissolved in 1 mL of ethanol. BMPA-capped MFN were added to the ethanol solution containing mPEG-AM, which was then stirred overnight at room temperature. Subsequently, PEGylated MFN were retrieved by centrifugation at 11,000 rpm for 15 min and finally dispersed in deionized water. For the characterization of MFN, the morphology of the NPs was analyzed with TEM using a JEOL EM-2010 microscope (JEOL, Tokyo, Japan) operated at 200 kV. The crystal structure of MFN was analyzed via XRD using a Bruker DE/D8 Advance system (Bruker, Karlsruhe, Germany). The metal ion concentration was measured with inductively coupled plasma atomic emission spectroscopy (ICP-AES) using an ICPS-8100 spectrometer (Shimadzu, Kyoto, Japan). The hydrodynamic sizes and zeta potentials were measured with DLS (Nano-ZS90, Malvern Panalytical, Malvern, UK).

### 4.2. Analysis of O_2_ Generation via H_2_O_2_ Decomposition

To demonstrate the Fenton catalytic effect of MFN, 2.5 mM of H_2_O_2_ and 2.5 mM of MFN were mixed in phosphate-buffered saline (PBS). Then, 150 µL of the solution was added to 300 µL of a Ti(SO_4_)_2_ solution (1.33 mL of 24% Ti(SO_4_)_2_ + 8.33 mL of H_2_SO_4_ in 50 mL of deionized water) every 10 min. The concentration of H_2_O_2_ was evaluated by measuring the absorbance at 408 nm using a UV-2600 spectrophotometer (Shimadzu). To investigate the capability of MFN to produce oxygen, dissolved oxygen was removed by bubbling the solution with argon. Subsequently, 0.5 mM of H_2_O_2_ was incubated with 0.5 mM of MFN in PBS. The O_2_ concentration was monitored using a dissolved oxygen meter (HI9146, HANNA Instruments, Woonsocket, RI, USA) every 5 min.

### 4.3. Cell Culture, Design of Hypoxic Environment, and Irradiation

Hepa1-6 mouse hepatoma cells were purchased from the Korean Cell Line Bank (Seoul National University, Seoul, Korea). Cells were cultured in Dulbecco’s modified Eagle’s medium (DMEM) supplemented with 10% fetal bovine serum (FBS, Sigma-Aldrich), 100 U/mL of penicillin (Sigma-Aldrich), 100 μg/mL of streptomycin (Sigma-Aldrich), and 25 mM HEPES (Gibco, Carlsbad, CA, USA). The cultures were maintained in a humidified atmosphere, containing 95% air and 5% CO_2_, at 37 °C. A hypoxic environment for irradiation was designed to minimize radiation scattering and the air gap [10]. Cells were placed in a hypoxia incubator chamber that was flushed with a gas mixture of 0.1% O_2_, 5% CO_2_, and 94.9 % N_2_ (Danil Syschem, Seoul, Korea). After gas flushing, the chamber was kept in an incubator at 37 °C in an atmosphere of 5% CO_2_ prior to irradiation. The cells placed in the chamber without gas flushing were used as the normoxic control. For IR treatment, 6 Mega Voltage (MV) photons at a rate of 3.96 Gy per minute from a Varian Clinac 6EX accelerator (Varian Medical Systems, Palo Alto, CA, USA) were used to irradiate cell monolayers.

### 4.4. Cell Viability Assay

The effects of MFN and IR on cell viability were determined using the CCK-8 colorimetric assay (Dojindo, Mashiki-machi, Japan). Hepa1-6 cells were seeded in 96-well plates, at a density of 5 × 10^3^ cells per well, for 12 h. After incubation with different doses of MFN, ranging from 0 to 50 μg/mL, for 24, 48, and 72 h, 10 μL of the CCK-8 solution was added to the wells and the plates were then incubated for another 2 h. The absorbance at 450 nm was measured using a SpectraMAX i3 microplate reader (Molecular Devices, San Jose, CA, USA). Relative cell viability was expressed as a percentage of the viability of the untreated control cells.

### 4.5. Clonogenic Survival Assay

A clonogenic survival assay was performed to determine the radiosensitivity of tumors under various treatments [10,11,39]. Hepa1-6 cells (200–1000 per well) were seeded in six-well plates. The cells were exposed to 0.1% oxygen (hypoxic conditions) or to normoxic conditions, and treated with MFN (5 or 10 μg/mL) for 3 h; next, the cells were exposed to 6 Gy of X-ray radiation. After 7 days, the cells were fixed in 98% ethanol (Sigma-Aldrich) and stained with 0.5% crystal violet (Sigma-Aldrich). Colonies containing more than 50 cells were counted using an inverted microscope (Zeiss Primovert; Carl Zeiss Co., Ltd., Jena, Germany).

### 4.6. Apoptosis Assay

Hepa1-6 cells (1 × 10^5^) plated in six-well plates were exposed to hypoxic and normoxic conditions with 5 μg/mL of MFN for 3 h, and then exposed to 6 Gy of X-ray radiation. After 72 h of irradiation, the extent of the apoptosis was evaluated with Annexin V-FITC staining and flow cytometry. First, the cells were trypsinized and washed with PBS (pH 7.4). This step was followed by staining with Annexin V-FITC (BD Biosciences, San Diego, CA, USA) and 2 μg/mL of propidium iodide in 100 μL of Annexin V binding buffer (10 mM HEPES, pH 7.4/140 mM NaCl/2.5 mM CaCl_2_) for 15 min at 37 °C in the dark. The population of apoptotic cells was determined by flow cytometry using a BD FACSVerse™ flow cytometer (BD Biosciences) and the BD FACSuite™ software (BD Biosciences).

### 4.7. HIF-1 and PD-L1 Expression

Hepa1-6 cells (2 × 10^5^) were plated in six-well plates and allowed to attach overnight. The cells were treated with 5 μg/mL of MFN for 3 h and subsequently exposed to IR under normoxic or hypoxic conditions. At 24 h after irradiation, the cells were collected by trypsinization and fixed in 4% formaldehyde (Sigma-Aldrich) for 10 min, and then permeabilized with 0.01% Triton X-100 (Sigma-Aldrich) for 3 min. Blocking was performed with 2% FBS in PBS for 30 min at room temperature, followed by incubation for 1 h at 25 °C with anti-HIF-1α (Novus Biologicals, Littleton, CO, USA), anti-γH2AX (Cell Signaling Technology, Danvers, MA, USA, or Millipore, Burlington, MA, USA), or anti-PD-L1 (Bio X cell, Lebanon, NH, USA) primary antibodies. Then, secondary antibodies were added, and cells were further incubated for 30 min. The samples were analyzed with flow cytometry using a BD FACSVerse™ instrument and the BD FACSuite™ software. Negative control staining was performed with secondary antibodies alone.

### 4.8. Cell Imaging of Immunofluorescence

For immunofluorescence microscopy, Hepa1-6 cells were seeded on a cover glass (Paul Marienfeld GmbH & Co. KG, Lauda-Königshofen, Germany) in a 12-well plate, fixed with 4% formaldehyde, and subsequently permeabilized using 0.01% Triton X-100. After blocking with 2% FBS for 30 min, the cells were incubated with anti-HIF-1α (Novus Biologicals) or anti-γH2AX (Cell Signaling Technology) primary antibodies, or with Alexa-Fluor488-conjugated phalloidin (Life Technologies, Eugene, OR, USA) for 1 h; next, the cells were incubated with DAPI (Sigma-Aldrich) and Alexa-Fluor-488-conjugated secondary antibodies for 30 min. Anti-rabbit and anti-mouse Alexa-Fluor-488-conjugated secondary antibodies were obtained from Molecular Probes (Carlsbad, CA, USA). The cells were washed, mounted using a fluorescent mounting medium (Dako, Carpinteria, CA, USA), and analyzed with fluorescent microscopy (Zeiss Observer D1; Carl Zeiss Co., Ltd., Seoul, Korea).

### 4.9. Animal Model

The animal experiments were reviewed and approved by the Institutional Animal Care and Use Committee of the Samsung Biomedical Research Institute (No. 20191203003). Six- to seven-week-old male C57BL6 mice were purchased from Orient Bio (Gapyeong, Korea). Hepa1-6 cells (1 × 10^6^ cells per 50 μL of PBS) were injected subcutaneously into the right hind leg. Tumor volumes were measured every 3 days, with calipers, and calculated according to the following formula: Volume = ((short diameter)^2^ × (long diameter))/2. When the tumor volume reached between 80 and 150 mm^3^, the mice were randomized into four groups as follows: (i) sham control (*n* = 7); (ii) MFN (*n* = 7); (iii) IR (*n* = 7); and (iv) MNF + IR (*n* = 7). Two hundred nanograms of MFN in 50 µL of PBS was intratumorally injected weekly, beginning on the day of randomization. The injections were continued until euthanasia. Four hours after the first injection, 8 Gy of X-ray irradiation was administered to the tumor in the right hind leg. During irradiation, the mice were anesthetized by injecting 30 mg/kg of tiletamine/zolazepam (Zoletil^®^, Virbac, Carros, France) and 10 mg/kg of xylazine (Rompun^®^, Bayer, Leverkusen, Germany) intraperitoneally following the prescription of a veterinarian. The mice were housed under barrier conditions and fed a standard rodent diet and water. At the end of the experiment, tumor tissues were fixed with 10% neutral buffered formalin (NBF; Sigma-Aldrich) and embedded in paraffin for IHC analysis.

### 4.10. TUNEL Assay

TUNEL assay was performed to estimate the rate of apoptosis in tumor tissues. The tissues were fixed with 10% NBF for 4 h and embedded in paraffin. Slices of tissues were deparaffinized in water and placed in 3% H_2_O_2_ for 10 min at room temperature. TUNEL staining was performed using the ApopTag^®^ Peroxidase In Situ Apoptosis Detection Kit (Millipore). Images were captured using an Aperio ScanScope AT instrument (Leica Biosystems, Buffalo Grove, IL, USA). The ratio of TUNEL-positive cells in the images was determined using the ImageScope software (Leica Biosystems).

### 4.11. Hypoxia Study and IHC Analysis

Pimonidazole (1-([2-hydroxy-3-piperidinyl]-propyl)-2-nitroimidazole) (Hypoxyprobe Inc., Burlington, MA, USA) was intraperitoneally injected at a dose of 60 mg/kg body weight 1 h before the animals were euthanized [18]. The tumor tissues were harvested immediately after euthanasia, fixed in 10% NBF buffer, and embedded in paraffin. Tissue sections were deparaffinized thrice in xylene for a total of 15 min and subsequently rehydrated. Immunostaining was performed using the Bond-MaxTM polymer refine detection kit (Vision Biosystems, Melbourne, Australia). Briefly, antigen retrieval was performed at 97 °C for 20 min in ER1 buffer. After blocking the endogenous peroxidase activity with 3% hydrogen peroxidase for 10 min, the samples were treated with anti-pimonidazole (Hypoxyprobe Inc.) and anti-PD-L1 (Bio X cell, Lebanon, NH, USA) primary antibodies for 15 min at room temperature.

### 4.12. Flow Cytometry for PD-L1 and T Cells

After measuring the tumor weight, excised tumors were dissociated into single cells using a gentleMACSTM Dissociator (Miltenui Biotec Inc., San Diego, CA, USA) with a mouse Tumor Dissociation kit (Miltenui Biotec, Bergisch Gladbach, Germany). The digested tissues were mashed through cell strainers (70 µm), and then red blood cells were removed. The harvested single cells were fixed with CytofixTM fixation buffer (BD Biosciences). The cells were washed with 1× PBS and centrifuged at 300× *g* for 5 min. They were then resuspended in BD PharmingenTM stain buffer (BD Biosciences). Next, the single-cell suspensions were incubated with the specific fluorescence-conjugated antibodies APC-conjugated CD45, V500-conjugated CD3, APC-Cy7-conjugated CD4, V450-conjugated CD8, and PE-conjugated PD-L1 (eBioscience, San Diego, CA, USA) at room temperature for 30 min in the dark. For intracellular staining, cells were fixed and permeabilized with Fixation/Permeabilization buffer (eBioscience) and stained with PE-Cy7-conjugated anti-IFNγ antibodies (BD Biosciences). All samples were evaluated using a BD FACSVerse™ system, and data analysis was performed using the FlowJo 10.6.2 software (BD Biosciences).

### 4.13. Statistical Analysis

Multiple groups were compared using one-way ANOVA with Tukey’s multi-comparison test using GraphPad Prism 7 (GraphPad Software, La Jolla, CA, USA). Tumor growth curves were analyzed using two-way ANOVA with Tukey’s correction. The statistical details of each experiment are indicated in the corresponding figure legends.

## 5. Conclusions

MFN were demonstrated to be effective radiosensitizers for Hepa1-6 hepatocellular carcinoma, alleviating hypoxic conditions in vitro and in vivo. MFN favorably regulated PD-L1 expression and the infiltration of T cells even after irradiation. We suggest that, combined with RT, MFN might have the potential to overcome therapeutic resistance in aggressive tumors.

## Data Availability

The data that support the findings of this study are available upon request to the corresponding authors.
